# Supplementary material for: Long working hours are associated with a higher risk of non-alcoholic fatty liver disease: A large population-based Korean cohort study
Source: PLoS One. 2021 Jul 23;16(7):e0255118. doi: 10.1371/journal.pone.0255118 (PMC8301658; doi:10.1371/journal.pone.0255118)
Supplement: S1 Table — (DOCX) [file pone.0255118.s001.docx]

| **S1 Table. Development of NAFLD according to weekly working hours among participants with no changes of working hours (n=30,311)** | | | | | | | |
| --- | --- | --- | --- | --- | --- | --- | --- |
| Weekly working hours | Person-years (PY) | Incident cases | Incidence density  (per 10^2^ PY) (95% CI) | Multivariable-adjusted HR (95% CI)^a^ | | | HR (95% CI)^b^ in model using time-dependent variables |
|  |  |  |  | Model 1^*^ | Model 2^**^ | Model 3^***^ |  |
| 35-40 | 33185.3 | 1532 | 4.62 (4.39-4.85) | 1.00 (reference) | 1.00 (reference) | 1.00 (reference) | 1.00 (reference) |
| 41-52 | 50238.2 | 3792 | 7.55 (7.31-7.79) | 1.20 (1.12-1.28) | 1.20 (1.12-1.28) | 1.21 (1.12-1.31) | 1.19 (1.10-1.29) |
| 53-60 | 3604.2 | 552 | 15.32 (14.09-16.65) | 2.54 (2.30-2.82) | 2.47 (2.23-2.74) | 2.54 (2.26-2.85) | 2.57 (2.29-2.89) |
| >60 | 1499.3 | 237 | 15.80 (13.92-17.95) | 2.73 (2.38-3.15) | 2.69 (2.33-3.11) | 2.90 (2.47-3.41) | 3.00 (2.56-3.52) |
| P for trend |  |  |  | <0.001 | <0.001 | <0.001 | <0.001 |
| ^a^ Estimated from Cox proportional hazard models. | | | | | | | |
| ^b^ Estimated from Cox proportional hazard models with alcohol intake, smoking status, regular exercise, BMI, hsCRP and HOMA-IR as time-dependent variables and baseline age, sex, center, year of screening exam, education level, weekly working hours, history of diabetes, medication for diabetes, history of hypertension, medication for hypertension and medication for dyslipidemia as time-fixed variables | | | | | | | |
| ^*^ Model 1 was adjusted for age, sex, center, and year of screening examination. | | | | | | | |
| ^**^ Model 2: model 1 plus adjustment for smoking status, alcohol intake, regular exercise, education level, history of diabetes, medication for diabetes, history of hypertension, medication for hypertension and medication for dyslipidemia. | | | | | | | |
| ^***^ Model 3: model 2 plus adjustment for BMI, hsCRP and HOMA-IR | | | | | | | |
